# High-Performance Regular Perovskite Solar Cells Employing Low-Cost Poly(ethylenedioxythiophene) as a Hole-Transporting Material

**DOI:** 10.1038/srep42564

**Published:** 2017-02-13

**Authors:** Xiaoqing Jiang, Ze Yu, Yuchen Zhang, Jianbo Lai, Jiajia Li, Gagik G. Gurzadyan, Xichuan Yang, Licheng Sun

**Affiliations:** 1 State Key Laboratory of Fine Chemicals, Institute of Artificial Photosynthesis, DUT-KTH Joint Education and Research Center on Molecular Devices, Dalian University of Technology (DUT), Dalian 116024, China; 2 Department of Chemistry, School of Chemical Science and Engineering, KTH Royal Institute of Technology, 100 44 Stockholm, Sweden

## Abstract

Herein, we successfully applied a facile *in-situ* solid-state synthesis of conducting polymer poly(3,4-ethylenedioxythiophene) (PEDOT) as a HTM, directly on top of the perovskite layer, in conventional mesoscopic perovskite solar cells (PSCs) (n-i-p structure). The fabrication of the PEDOT film only involved a very simple *in-situ* solid-state polymerisation step from a monomer 2,5-dibromo-3,4-ethylenedioxythiophene (DBEDOT) made from a commercially available and cheap starting material. The ultraviolet photoelectron spectroscopy (UPS) demonstrated that the as-prepared PEDOT film possesses the highest occupied molecular orbital (HOMO) energy level of −5.5 eV, which facilitates an effective hole extraction from the perovskite absorber as confirmed by the photoluminescence measurements. Optimised PSC devices employing this polymeric HTM in combination with a low-cost vacuum-free carbon cathode (replacing the gold), show an excellent power conversion efficiency (PCE) of 17.0% measured at 100 mW cm^−2^ illumination (AM 1.5G), with an open-circuit voltage (*V*_*oc*_) of 1.05 V, a short-circuit current density (*J*_*sc*_) of 23.5 mA/cm^2^ and a fill factor (*FF*) of 0.69, respectively. The present finding highlights the potential application of PEDOT made from solid-state polymerisation as a HTM for cost-effective and highly efficient PSCs.

Recently, organic-inorganic halide perovskites with the composition ABX_3_ [A = CH_3_NH_3_^+^ (MA), NH = CHNH_3_^+^ (FA) or Cs^+^; B = Pb or Sn; X = Cl, Br, I] have received increasing research attention as light absorbers in solid-state thin-film solar cells^1^,^2^,^3^,^4^,^5^,^6^,^7^. The power conversion efficiency (PCE) of perovskite solar cells (PSCs) has been rapidly increased to over 22% in less than four years^8^. The high efficiency together with facile fabrication routes make PSCs the forerunner among the low-cost next generation solar cell technologies.

The state-of-the-art high-performing PSC devices routinely incorporated hole-transporting materials (HTMs) as p-type contacts, which play a key role in extraction and collection of the photo-generated holes from the perovskites, and thus minimizing undesired recombination losses at the interfaces^9^. A broad range of HTMs have been developed and incorporated in PSCs, which are composed of organic hole-conductors and inorganic p-type semiconductors. Altough inorganic p-type semiconductors (copper iodide and copper thiocyanate) possess some superior advantages, i.e. high hole mobility and ease of synthesis, the PCEs of PSCs based on these HTMs were not satisfactory^10^,^11^,^12^, with the highest reported value of 16.6%^13^. Organic HTMs mainly consist of small molecule hole-conductors and conducting polymers. A large number of small molecule HTMs have been synthesized and applied in PSCs, including triphenylamine (TPA)-based, carbazole-based, donor-acceptor (D-A) conjugated small molecules, tetrathiafulvalene and pentacene derivatives etc.^14^,^15^,^16^,^17^,^18^,^19^,^20^,^21^,^22^,^23^,^24^,^25^ Among them, spiro-type small molecule HTMs, such as 2,2′,7,7′-tetrakis(N,N-di-p-methoxyphenylamine)-9,9′-spirobifluorene (spiro-OMeTAD), have exhibited the superior overall efficiency^15^,^23^, with the highest PCE of over 20%^26^.

Conducting polymers constitute another major class of organic HTMs in PSCs. A number of conducting polymers have been used as HTMs in PSCs. Polythiophene-based conducting polymer P3HT (poly(3-hexylthiophene-2,5-diyl)), which has been widely used as model hole conductors in organic solar cells and organic field-effect transistors, were intensively studied in PSCs^27^,^28^,^29^,^30^,^31^,^32^. However, the devices based on P3HT HTMs typically exhibited mediocre performance. P3HT functionalized with highly conductive carbon materials (graphdiyne) showed an improved overall efficiencies, with a maximum PCE of 14.58%^31^. Other type of thiophene-based conducting polymers have also been tested as HTMs in PSCs, exhibiting poor efficiencies below 10%^27^,^33^,^34^,^35^. Polyfluorene derivatives also showed unsatisfactory performance with the highest efficiency of only 12.8%^36^. High overall efficiencies of PSC devices based on polymeric HTMs have been rarely reported thus far. Very recently, Park and co-workers developed a donor-acceptor based conducting polymer as a HTM in planar PSCs, showing a maximum PCE of 17.3% without using lithium salt additives^37^. Poly(triarylamie) (PTAA) has been the only example of polymeric HTMs used in PSCs that could work as efficiently as spiro-type small molecules^38^,^39^,^40^. The highest reported PCE of PSCs based on PTAA also reached more than 20%^39^. However, the high-performing polymeric HTMs reported so far typically require complicated synthesis, especially for the monomers (tedious synthesis and high purity required), thus resulting in high production costs. For instance, the price of the best polymeric HTM PTAA is extremely expensive (50 times higher than gold), which significantly impedes its large-scale application in the future^41^. There is therefore a good motivation to explore alternative polymeric HTMs combining high photovoltaic performance together with low production costs.

Poly(3,4-ethylenedioxythiophene) (PEDOT) is a cheap conducting polymer with high conductivity and has been widely used in both organic photovoltaics and light emitting diodes as a charge collection or charge injection layer^42^,^43^. However, PEDOT is typically heavily doped by being stabilized with polystyrenesulfonate (PSS) into an aqueous dispersion^36^. PEDOT:PSS has been widely used as HTMs in inverted PSCs (p-i-n), where the perovskite layers were deposited on top of the PEDOT:PSS^44^. Yet, the direct deposition of PEDOT:PSS on top of the perovskite film seems to be quite challenging, because moisture is detrimental to the perovskites. Therefore, the use of PEDOT as HTMs in conventional PSC structures (n-i-p) has been rarely reported so far. Snaith *et al*. employed a specifically formulated PEDOT ink, which is a dispersion of PEDOT and a sulfonated block-co-polymer dispersed in toluene, as a HTM in PSCs with a cell configuration of FTO glass/TiO_2_ compact layer/mesoporous Al_2_O_3_ + MAPbI_3−x_Cl_x_/PEDOT/Au^36^. The PSC devices using this low-cost HTM achieved PCE of up to 14.5% under one sun illumination, which performed as well as the commonly used spiro-OMeTAD. However, the stabilized power output was found to be lower for PEDOT-based cells (6.6%).

Herein, we report facile solid-state synthesis of conducting polymer PEDOT as a HTM in mesoporous PSCs based on a (FAPbI_3_)_0.85_(MAPbBr_3_)_0.15_ light absorber. The PEDOT film was fabricated straightforwardly on top of the perovskite layer through an *in-situ* solid-state polymerisation from an inexpensive monomer 2,5-dibromo-3,4-ethylenedioxythiophene (DBEDOT). PSCs employing this polymeric HTM show an impressive PCE of 17.0% measured at 100 mW cm^−2^ illumination (AM 1.5G), which is one of the highest reported value for conducting polymer-based HTMs. The present finding highlights the potential application of PEDOT made from solid-state synthesis as a HTM for cost-effective and high-performing PSCs.

## Results

**Figure 1:**
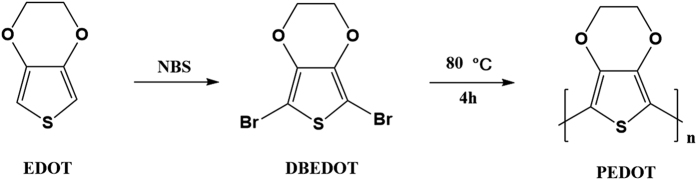
Solid-state synthesis of PEDOT, formed from heating monomer DBEDOT at 80 °C for 4 h.

**Figure 2:**
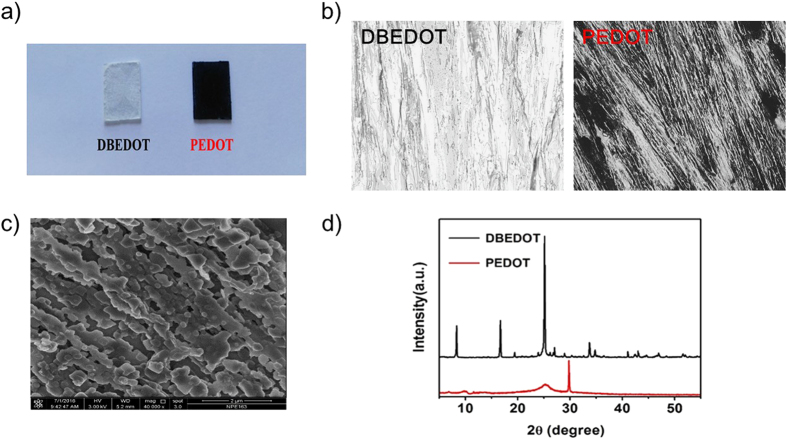
(**a**) Photographs of monomer DBEDOT and PEDOT prepared on glass substrates. (**b**) Optical microscopy images of monomer DBEDOT (left) and PEDOT (right) formed from solid-state synthesis (magnification 200x). (**c**) SEM image of the surface of PEDOT fabricated from solid-state synthesis. (**d**) XRD spectra of monomer DBEDOT (black) and PEDOT fabricated from *in-situ* polymerisation method (red) on glass substrates.

DBEDOT (Fig. 1) was chosen as the solid-state polymerisable monomer for the synthesis of conducting polymer PEDOT. DBEDOT was synthesized *via* a common bromination method from a commercially available and cheap starting material 3,4-ethylenedioxythiophene (EDOT) as reported previously^45^, which was confirmed by ^1^H NMR spectroscopy (Figure S1, Supporting Information (SI)). Conducting polymer PEDOT was subsequently prepared through an *in-situ* solid-state polymerisation of DBEDOT analogous to a reported method^45^,^46^. The schematic solid-state synthesis route is presented in Fig. 1. Monomer DBEDOT was incubated at 80 °C for 4 h in a closed vial, during which period the color of the material changed from white to dark blue in the state of thin films (Fig. 2a). It indicates that the annealing process facilitated the transformation of monomer DBEDOT to polymer PEDOT. In Fig. 2b, the optical microscopy images clearly show that the colorless needle-shaped crystals of DBEDOT was transformed to black crystals of PEDOT. Scanning electron microscopy (SEM) image of the surface of PEDOT fabricated from solid-state synthesis is presented in Fig. 2c. The transformation of DBEDOT to polymer PEDOT was further confirmed by x-ray diffraction (XRD), as shown in Fig. 2d. The crystalline DBEDOT thin film shows main diffraction peaks at 2θ = 8.5, 16.8, 25.4, 27.0 and 34°. By stark contrast, a strong and sharp (line width *ca.* 0.2°) diffraction peak at 2θ = 30° was observed for the PEDOT thin film sample, which obviously do not belong to the DBEDOT and must be attributed to the structure of the formed polymer^45^.

**Figure 3:**
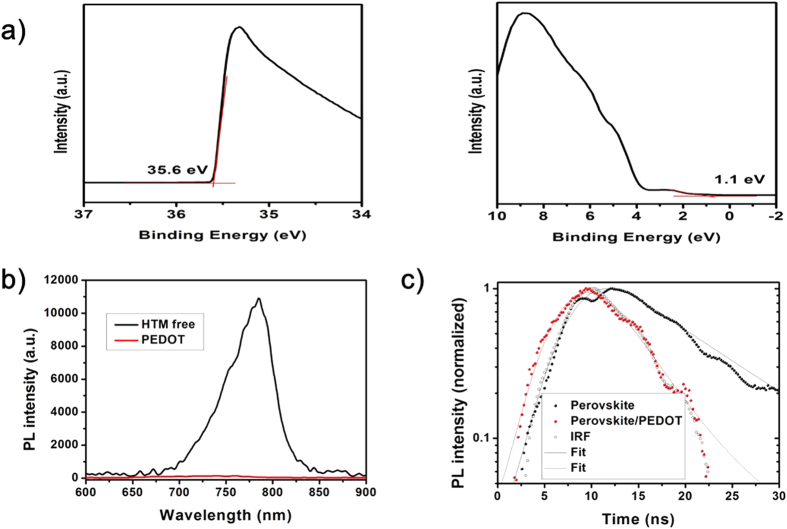
(**a**) UPS spectra in the cutoff (left) and onset (right) energy regions for PEDOT film from solid-state synthesis. (**b**) Steady-state PL of glass/perovskite films with (red) and without (black) PEDOT at excitation wavelength 532 nm. (**c**) Decay kinetics of PL of glass/perovskite films with (red) and without (black) PEDOT monitored at 780 nm after excitation with 532 nm. Open circles: instrument response function (IRF).

Effective hole extraction from the perovskite absorber is primarily important for a HTM to function well in PSCs. The highest occupied molecular orbital (HOMO) energy level of PEDOT fabricated from solid-state synthesis was measured using ultraviolet photoelectron spectroscopy (UPS). Figure 3a display the cutoff (E_cutoff_) (left) and onset (E_i_) (right) energy regions in the UPS spectrum for the as-prepared PEDOT film, respectively. The HOMO energy level of PEDOT is calculated to be −5.5 eV, according to the equation *φ* = 40 − (E_cutoff_ − E_i_). Therefore, the HOMO energy level of as-prepared PEDOT matches well with the valence band (VB) of perovskites (FAPbI_3_)_0.85_(MAPbBr_3_)_0.15_ (−5.65 eV)^47^,^48^. Thus, PEDOT should be capable of effectively extracting holes from the perovskite (FAPbI_3_)_0.85_(MAPbBr_3_)_0.15_. To verify this hypothesis, we carried out steady-state photoluminescence (PL) and time-resolved PL decay characteristics of perovskite/PEDOT films. From the steady-state PL spectroscopy as shown in Fig. 3b, a significant quenching effect, factor of 80 (ratio of areas of PL intensity of HTM free and PEDOT samples), was observed in the presence of PEDOT. In order to get further insight on the quenching, we have also performed PL kinetic measurements *via* monitoring at the emission maximum of 780 nm (Fig. 3c). PL in perovskite decays with a lifetime of 10 ns, whereas the corresponding value in perovskite with PEDOT is much shorter. Instrument response function (IRF) of our setup was about 2 ns, which allows by using deconvolution/fit procedure to get the time-resolution of 1 ns (see Methods). In PEDOT, the decay kinetics was close to the IRF (Fig. 3c), i.e. beyond the time-resolution of our setup. Therefore we can only estimate the upper limit of the lifetime to be 1 ns. However, we can also calculate the PL lifetime in perovskite/PEDOT from equation: *τ*_*f*_ = *τ*_*rad*_*φ*_*f*_, where τ_f_ is the lifetime of fluorescence, τ_rad_ is the radiative (native) lifetime and φ_f_ is the quantum yield of fluorescence. Under the assumption of constant τ_rad_ in HTM free and PEDOT, factor of 80 quenching of fluorescence (Fig. 3b) will lead in shortening of the fluorescence lifetime from 10 ns to 125 ps. A shorter lifetime in the presence of PEDOT is indicative for efficient charge transfer at the interface of perovskite/PEDOT as a result of hole extraction. Thus, PEDOT can be considered as a highly efficient hole acceptor for the perovskite absorber studied in this work. Once extracted from the perovskite layer, the holes must be quickly transported to the cathode. Thus, the conductivity of the PEDOT film fabricated from the solid-state synthesis was tested by four-point probe measurement. The conductivity of as-prepared PEDOT film (three samples on average) is quite high, amounting to be *~*5.3 × 10^−1^ S cm^−1^. Based on these results discussed above, we can conclude that PEDOT demonstated in this work could effectively extact and collect the photo-generated holes from the perovskite absorber layer.

**Figure 4:**
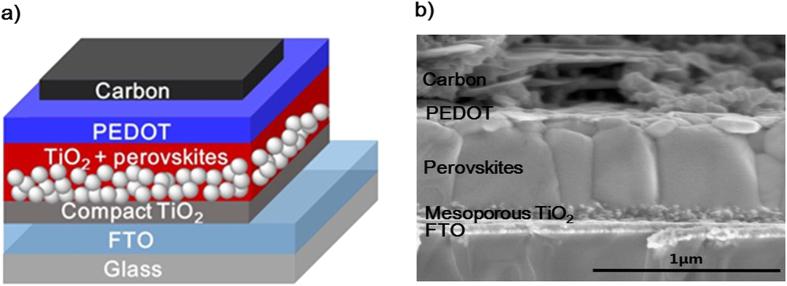
(**a**) Schematic device architecture of perovskite solar cells studied. (**b**) Cross-sectional SEM image of the complete PSC device containing FTO glass/compact TiO_2_/mesoporous TiO_2_/perovskite/PEDOT/Carbon.

We further applied PEDOT film fabricated from solid-state synthesis, as a HTM in mesoscopic PSCs. The schematic illustration and cross-sectional SEM image of the device architecture are depicted in Fig. 4a and b. The solar cell devices were fabricated with a structure of FTO glass/compact TiO_2_ (~30–40 nm)/mesoporous TiO_2_ (~150 nm)/perovskite/HTM/Carbon. The mixed-cations perovskite light absorber (FAPbI_3_)_0.85_(MAPbBr_3_)_0.15_ was prepared by using a solvent-engineering technique as reported previously by Seok *et al*.^40^. Perovskite crystals grew inside the pores of TiO_2_ scaffold and additionally formed a capping layer with a total thickness of about 600 nm. The XRD and UV-vis spectra of fabricated mixed cation perovskite are presented in Figs S2 and S3, respectively, in the SI. The resulting perovskite films were spin-coated with a layer of monomer DBEDOT. Finally, a low-cost vacuum free carbon cathode was deposited on top of the PEDOT layer by doctor-blading method as reported previously^49^,^50^,^51^. PEDOT HTM layer was formed in between perovskite absorber layer and carbon cathode.

**Figure 5:**
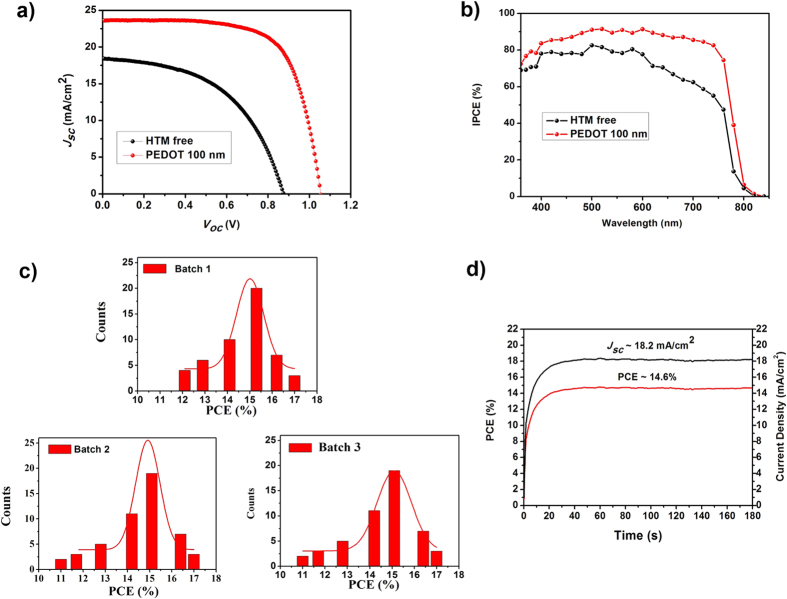
(**a**) *J*-*V* characteristics and (**b**) IPCE spectra of the PSC devices with PEDOT (100 nm) (red) and without HTM (black). (**c**) Histogram of PCEs of three batches of PSC devices (50 cells per batch) using PEDOT (100 nm) as HTMs. (**d**) PCE (red) and current density (black) as a function of time under illumination at a fixed voltage of 0.8 V for a representative device based on PEDOT.

A reference cell without any HTM was prepared for comparison, exhibiting a low PCE of only 8.5% with an open-circuit voltage (*V*_*oc*_) of 0.90 V, a short-circuit current density (*J*_*sc*_) of 18.4 mA/cm^2^ and a fill factor (*FF*) of 0.51, as shown in Fig. 5a and Table 1. The introduction of PEDOT as a HTM with vaired thicknesses (50, 100 and 200 nm) significantly improves the photovoltaic parameters (*V*_*oc*_, *J*_*sc*_ and *FF*), thus leading to much higher overall efficiencies of over 15% (Figure S4, SI). The incoporation of the PEDOT hole-transporting layer could substantially prevent direct contacts between the perovskite layer and carbon cathode, thus reducing the recombination rates and faciliating an effecitive hole extraction/collection in these devices. Under an optimal condition (~100 nm PEDOT), the best PSC device displays an impressive PCE of 17.0%, with a *V*_*oc*_ of 1.05 V, a *J*_*sc*_ of 23.5 mA/cm^2^ and a *FF* of 0.69, respectively (Fig. 5a). The photovoltaic performance of PSC devices based on the well-known HTM spiro-OMeTAD with additives 4-*tert*-butylpyridine (TBP) and lithium bis(trifluoromethanesulfonyl)imide (LiTFSI) was also studied in combination with both carbon and gold counter electrodes, as shown in Figure S5 and Table S1 in the SI. The best PSC devices display PCEs of 15.2% (carbon) and 18.6% (gold), respectively. Figure 5b presents the incident-photon-to-current conversion efficiency (IPCE) spectra for PSCs with and without PEDOT (~100 nm). Both of these two PSC devices display a wide spectra response to a long wavelength limit at around 840 nm. As compared to the device without HTM, the incorporation of PEDOT exhibits a remarkable improvement of IPCE over the whole region measured, yielding a broad IPCE plateau of over 85% in the range of 420 to 700 nm. Here, it emphasizes again that PEDOT is a suitable HTM that can effectively extract and collect the photo-generated holes from the perovskite.

The statistical data of three batches of PSC devices (50 cells per batch) employing PEDOT as HTMs are shown in Fig. 5c. The PCEs of PEDOT-based PSCs are highly reproducible from batch to batch, with 60% of the devices yielding PCEs of over 15%. We further carried out stabilized power output of PEDOT-based devices at a fixed voltage close to maximum power point on the *J*-*V* curve. It was reported that the stabilized power output is a reliable and scan-independent method to determine the efficiency of a PSC device^28^. Figure 5d displays the steady-state efficiencies of a representative PSC device based on PEDOT measured at a constant bias of 0.8 V over 180 seconds under 1 sun condition (AM 1.5G). PEDOT-based device exhibits a steady-state efficiency of 14.6% and current density of 18.2 mA/cm^2^ during the testing period. The stability of PSCs devices based on PEDOT, which were stored at ambient condition in the dark with a humidity of ~30%, was also investigated for 720 hours (Figure S6, SI). The devices remained over 80% of their initial PCEs during the aging time. The stability of PSCs devices based on spiro-OMeTAD with additives were also studied. The results are shown in Figure S7 (SI).

**Figure 6:**
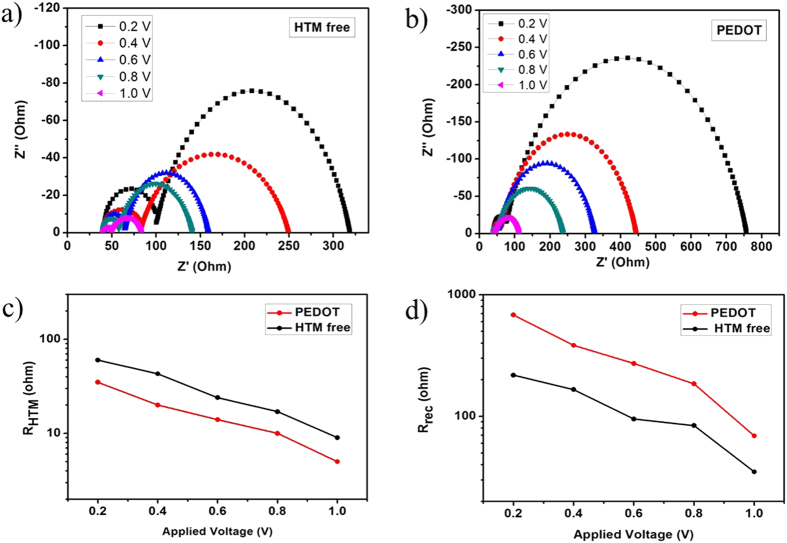
Nyquist plots of PSC devices without HTM (**a**) and with PEDOT (**b**) measured under one sun (one sun illumination) illumination with varied forward biases. (**c**) Hole-transport resistance and (**d**) charge recombination resistance extracted from EIS measurements at varied bias potentials.

Electrochemical impedance spectroscopy (EIS) was carried out to gain more insight into electrical characteristics of PSCs with and without PEDOT as HTMs under one sun illumination (AM 1.5 G, 100 mW · cm^−2^) with varied bias voltage in the frequency range from 10^6^ to 10^−1^ Hz. The Nyquist plots are displayed in Fig. 6a and b. According to a previously reported equivalent circuit model^52^,^53^ the corresponding results are plotted in Fig. 6c and d. The Nyquist plots exhibit two arcs: one arc in the high frequency region is associated to the hole transport and extraction between the HTM and the carbon cathode (*R*_*HTM*_); the main arc in the low frequency region represents the characteristics of charge recombination (*R*_*rec*_). From Fig. 6c, it is distinct that at a fixed bias potential PSC device employing PEDOT shows a lower *R*_*HTM*_ than the corresponding value for the device without any HTM, indicating a more sufficient hole transport/collection for the PEDOT-based device. This result accounts for the higher *FF* obtained for the device containing PEDOT as a HTM. The larger *R*_*rec*_ observed for the devices employing PEDOT as a HTM (Fig. 6d) could explain the higher *V*_*oc*_ achieved from the *J*-*V* measurements.

## Conclusion

A facile *in-situ* solid-state synthesis of conducting polymer poly(3,4-ethylenedioxythiophene) (PEDOT) has been successfully incorporated as a hole-transporting material in PSCs. Optimised PSC devices employing this polymeric HTM in combination with a mix-cation perovskite absorber and a non-Au vacuum free low-cost carbon cathode, show a maximum PCE of 17.0% measured at 100 mW cm^−2^ illumination (AM 1.5G), together with good reproducibility from batch to batch. More strikingly, the fabrication of the PEDOT film on top of the perovskite layer only involved a very simple *in-situ* solid-state polymerisation step from a monomer DBEDOT, which was made from a commercially available and cheap starting material. The simple processing of PEDOT presented in this work combined with low production cost and the high overall efficiency makes it a good candidate material for future application of perovskite solar cells where cost-effective and large-area manufacture is required.

## Methods

### Materials

All chemicals and solvents were used as received unless otherwise stated, including PbI_2_ (>98%, TCI), PbBr_2_ (99%, Sigma-Aldrich), HI (48% in water, Sigma-Aldrich), HBr (48% in water, Sigma-Aldrich), CH_3_NH_2_ (33 wt.% in absolute ethanol, Sigma-Aldrich), Formamidine acetate (99%, Sigma-Aldrich), Titanium diisopropoxide bis(acetylactonate) 75% in isopropanol (Tiacac, Sigma-Aldrich), mesoporous-TiO_2_ paste (18NR-T, Dyesol). NH_2_CH = NH_2_I (FAI) was synthesized according to a reported procedure^40^. 30 ml hydroiodic acid (57% in water) and 15 g formamidine acetate were reacted at 0 °C for 2 h with stirring. The precipitates were recovered by evaporating the solutions at 50 °C for 1 h. The products were dissolved in ethanol, recrystallized using diethyl ether, and finally dried at 60 °C in a vacuum oven for 24 h. Similarly, CH_3_NH_3_Br (MABr) were prepared by reacting hydrobromic acid (48 wt% in water) with methylamine according to a reported procedure^26^. The mixed-cation perovskite precursor solution of (FAPbI_3_)_1−x_(MAPbBr_3_)_x_ (x = 0.15) was prepared in a glovebox, by dissolving the FAI (1 M), MABr (0.2 M), PbI_2_ (1.1 M) and PbBr_2_ (0.2 M) in a mixed solvent of dimethyl formamide (DMF) and dimethyl sulfoxide (DMSO) (4:1, v/v).

2,5-Dibromo-3,4-ethylenedioxythiophene (DBEDOT) was prepared according to a previously reported method^45^. *N*-bromosuccinimide (0.202 mol) was added slowly to a stirred solution of 3,4-ethylenedioxythiophene (EDOT) (0.10 mol) dissolved in a 2:1 solvent mixture of chloroform (300 mL) and acetic acid (150 mL) at 0–5 °C under argon atmosphere. The mixture was allowed to stir for 8 h at room temperature and quenched with water. The organic layer was separated, and the water layer was extracted with chloroform (100 mL ×3). The combined chloroform extract was neutralized with 5% sodium bicarbonate solution, washed with distilled water, and dried with anhydrous magnesium sulfate. The filtered solution was concentrated, passed through a silica gel column, and eluted with methylene chloride to give a crude white powder. It was recrystallized from ethanol to produce white needlelike crystals in 75% yield. ^1^H NMR (500 Hz) (CDCl_3_), H 4.27 (s, 4 H) ppm.

### Device Fabrication and Characterization

Fluorine–doped tin oxide (FTO)-coated glass (Pilkington TEC 15) was firstly patterned by etching with Zn powder and 2 M HCl. The etched substrate was then sequentially cleaned by using detergent, de-ionized water and ethanol. Remaining organic residues were removed under oxygen plasma for 30 min. A compact TiO_2_ blocking layer (BL) of roughly 30–40 nm was deposited on the cleaned FTO glasses by spray pyrolysis of titanium diisopropoxide bis(acetylacetonate) diluted in anhydrous ethanol at a volumetric ratio of 1:10 and then heated at 500 °C for 30 min. A mesoporous TiO_2_ layer was deposited by spin-coating TiO_2_ paste (Dyesol 18NR–T) diluted in anhydrous ethanol at ratio of 1:5 by weight at 5000 rpm for 30 s. The layers were then sintered in air at 500 °C for 30 min. The mixed-cation perovskite films were deposited onto the mesoporous TiO_2_/BL TiO_2_/FTO substrate substrates from the precursor solution by a two-step spin-coating procedure, at 1000 rmp for 30 s and then 5000 rmp for 20 s. During the second step, 200 μL of chlorobenzene was dropped onto the substrates 10 s prior to the end of the program. Subsequently, the HTM PEDOT layer was produced by spin-coating the monomer DBEDOT with different concentrations (0–200 mg/mL in chlorobenzene) on top of the perovskite layer with a spin speed of 3000 rpm for 30 s. Finally, a commercial carbon paste (CC, Shenzhen DongDaLai Chemical Co., Ltd.) was deposited on top of the PEDOT layer by doctor-blading method as reported previously and dried the whole device at 80 °C for 4 h^49^,^51^.

The photocurrent–voltage (*J*-*V*) characteristics of the solar cells were measured using a Keithley 2400 Source-measure unit under illumination of a simulated sunlight (AM 1.5 G, 100 mW · cm^−2^) provided by an Oriel Sol3A solar simulator (Newport USA, Model: 94023A) with an AM 1.5 filter in ambient air. Light intensity was calibrated with a Newport calibrated standard Si reference cell (SER. No: 506/0358). A black mask with a circular aperture (0.09 cm^2^) smaller than the active area of the square solar cell (0.20 cm^2^) was applied on top of the cell. The *J*-*V* curves were obtained from forward bias to short-circuit at a scan rate of 10 mV S^−1^. The measurement of the incident photo–to–current conversion efficiency (IPCE) was obtained by a Hypermono–light (SM–25, Jasco Co. Ltd., Japan). Prior to measurement, a standard silicon solar cell was used as reference.

### Characterizations

^1^H NMR spectrum was recorded on a Bruker Ascend^TM^ 500 NMR apparatus. Chemical shifts were calibrated against TMS as an internal standard. XRD was implemented with Rigaku SmartLab (9). The measurement of microphotograph was obtained by Olympus BX51M (magnification, 200x). The measurement of conductivity was implemented by RTS-9 dual four-probe electrical measurement. The HOMO energy level of the PEDOT film was examined using ultraviolet photoelectron spectroscopy (UPS) with photon energy of 40 V. A sample bias of 5.0 V was applied to observe the secondary electron cutoff. SEM was performed with FEI (Field Emission Instruments: Nova Nano SEM 450), USA. Steady-state and time-resolved photoluminescence (PL) measurements were performed by use of nanosecond flash-photolysis system (LP920, Edinburgh Instruments) with excitation wavelength of 532 nm, pulse-width 5 ns, single shots. Instrument response function (IRF) was obtained by monitoring the time evolution of 532 nm excitation light scattered from the same perovskite sample. Single exponential fit/deconvolution was performed. Overall time resolution was about 1 ns. The electrochemical impedance spectroscopy (EIS) measurements were carried out at different applied bias under one sun illumination (AM 1.5 G, 100 mW · cm^−2^) using an impedance/gain–phase analyzer (Zahner Model: Zennium, Serial No: 40037, German) electrochemical workstation with the scanning frequency range from 10^6^ to 0.1 Hz. The magnitude of the alternative signal was 10 mV.

## Additional Information

**How to cite this article:** Jiang, X. *et al*. High-Performance Regular Perovskite Solar Cells Employing Low-Cost Poly(ethylenedioxythiophene) as a Hole-Transporting Material. *Sci. Rep.* **7**, 42564; doi: 10.1038/srep42564 (2017).

**Publisher's note:** Springer Nature remains neutral with regard to jurisdictional claims in published maps and institutional affiliations.

## Supplementary Material

Supplementary Information

## Figures and Tables

**Table 1:** Photovoltaic parameters of PSCs based on HTM PEDOT with different thicknesses measured under 100 mW cm^−2^ illumination (AM 1.5G).

Thickness of PEDOT (nm)	*V*_*oc*_ (V)	*J*_*sc*_ (mA/cm^2^)	*FF*	*PCE* (%)
0	0.90	18.4	0.51	8.5
50	1.03	23.3	0.67	16.1
100	1.05	23.5	0.69	17.0
200	1.02	23.6	0.66	15.9
