# Supplementary material for: High-Performance Regular Perovskite Solar Cells Employing Low-Cost Poly(ethylenedioxythiophene) as a Hole-Transporting Material
Source: Sci Rep. 2017 Feb 13;7:42564. doi: 10.1038/srep42564 (PMC5304166; doi:10.1038/srep42564)
Supplement: Supplementary Information [file srep42564-s1.doc]

**High-Performance Regular Perovskite Solar Cells Employing Low-Cost Poly(ethylenedioxythiophene) as a Hole-Transporting Material**

Xiaoqing Jiang^1^, Ze Yu^1^, Yuchen Zhang^1^, Jianbo Lai^1^, Jiajia Li^1^, Gagik G. Gurzadyan^1^, Xichuan Yang^1^ and Licheng Sun^1,2^

^1^State Key Laboratory of Fine Chemicals, Institute of Artificial Photosynthesis, DUT-KTH Joint Education and Research Center on Molecular Devices, Dalian University of Technology (DUT), Dalian 116024, China. ^2^Department of Chemistry, School of Chemical Science and Engineering, KTH Royal Institute of Technology, 100 44 Stockholm, Sweden. Correspondence and requests for materials should be addressed to Z.Y. ([ze.yu@dlut.edu.cn](mailto:ze.yu@dlut.edu.cn)) or L.S. (lichengs@kth.se)


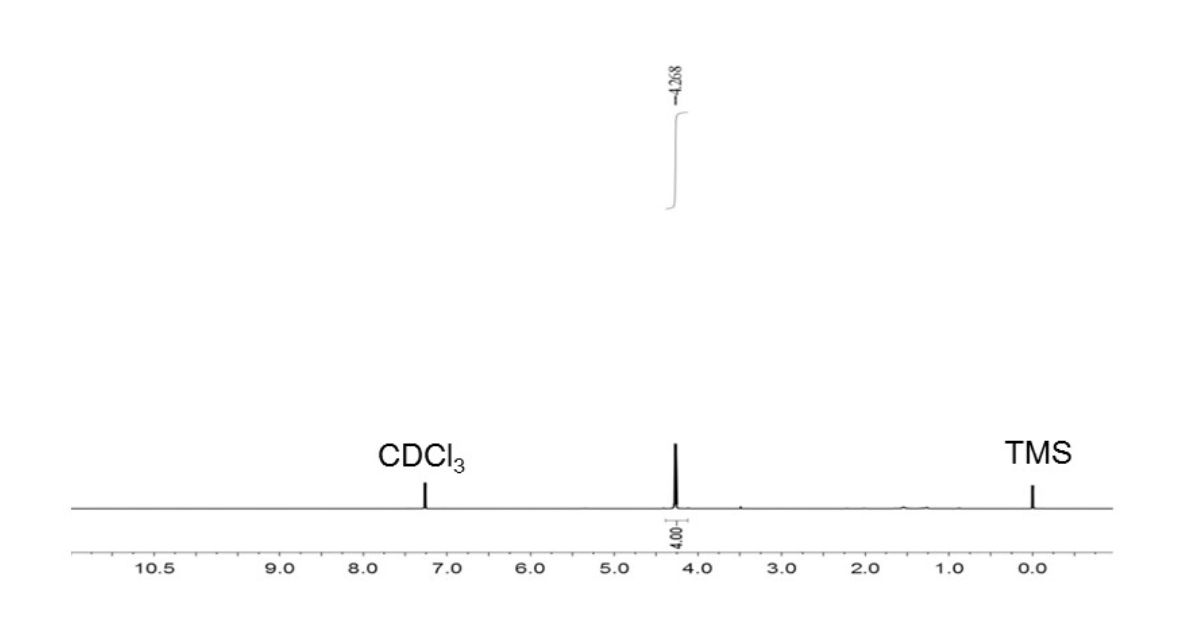


Figure S1. ^1^H NMR spectrum of compound DBEDOT.


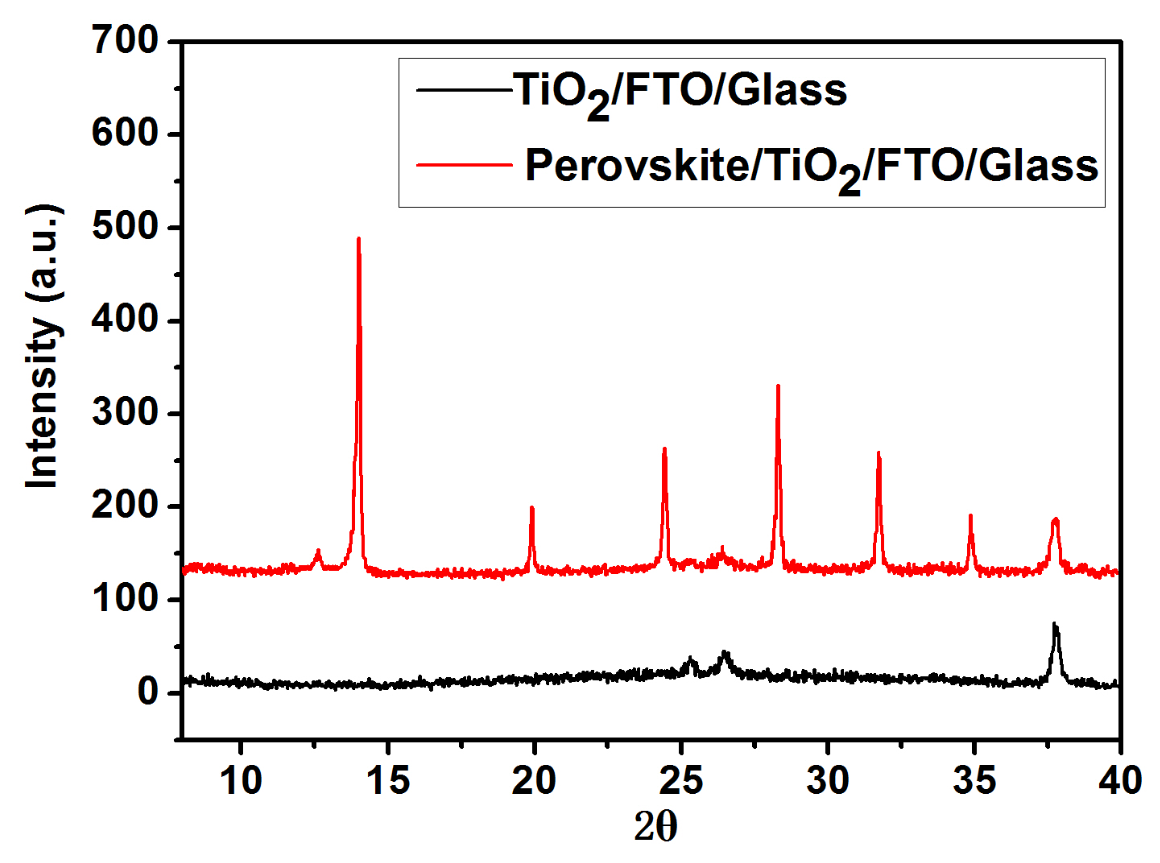


Figure S2. X–ray diffraction (XRD) patterns of mesoporous TiO_2_ on FTO substrate (black line) and (FAPbI_3_)_0.85_(MAPbBr_3_)_0.15_ on mesoporous TiO_2_/FTO film (red line).


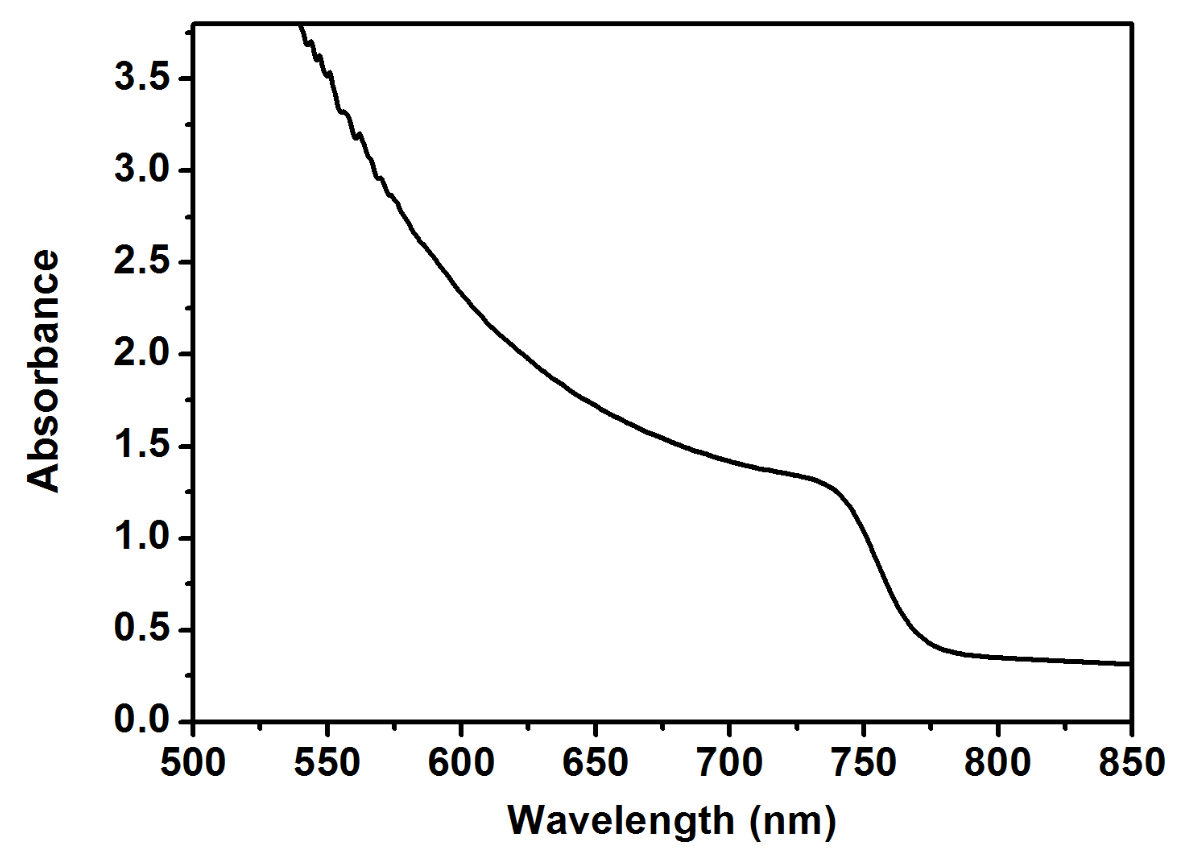


Figure S3. UV-vis absorption spectrum of (FAPbI_3_)_0.85_(MAPbBr_3_)_0.15_ on mesoporous TiO_2_/FTO substrate film.


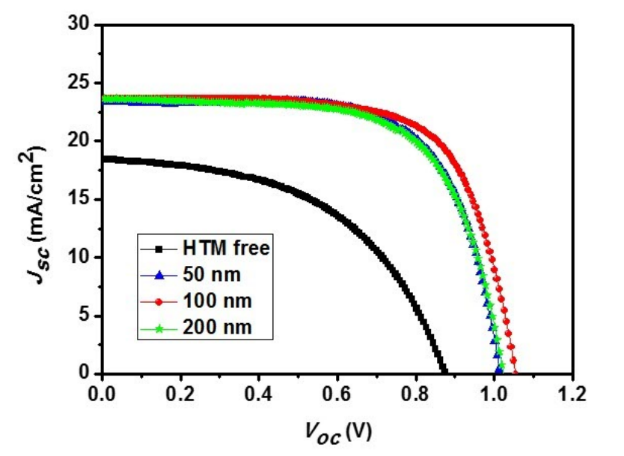


Figure S4. *J*-*V* characteristics of PSC devices without HTM and with different thicknesses of PEDOT.


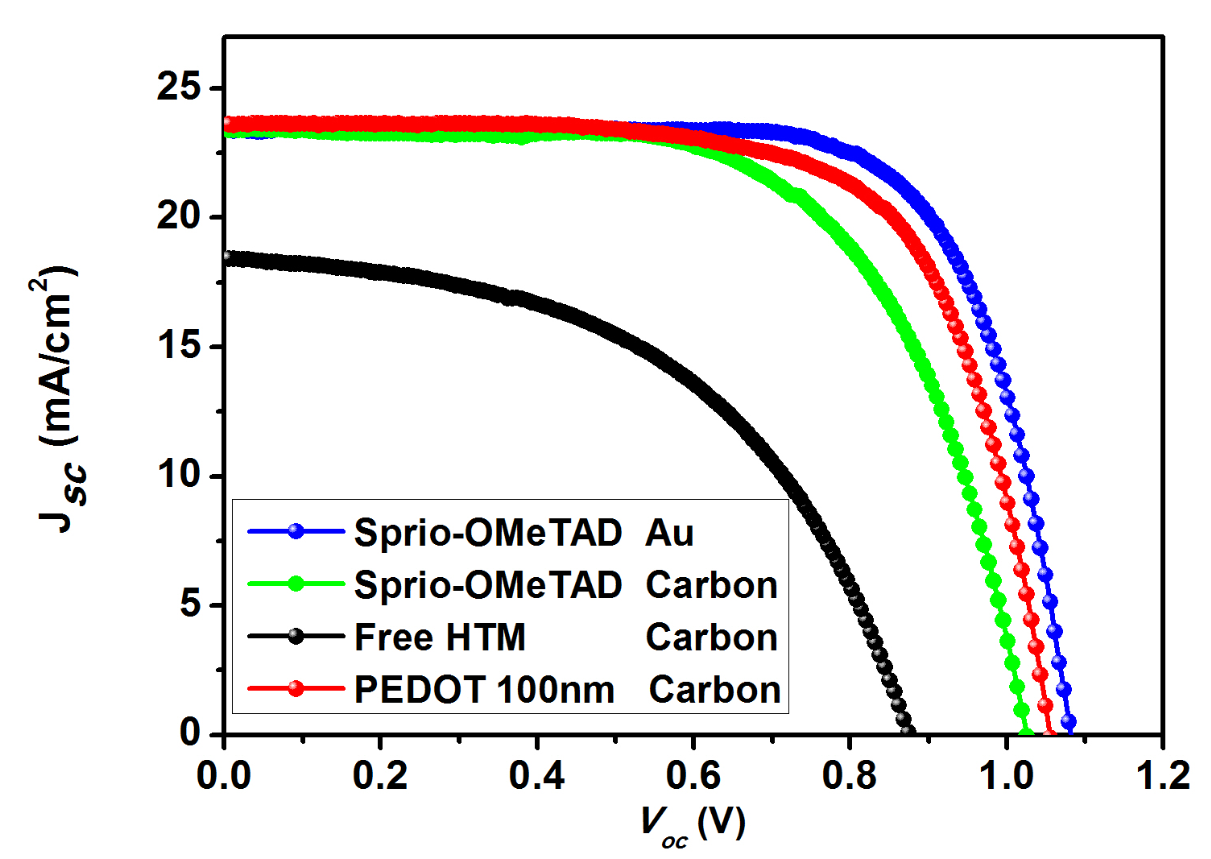


Figure S5. *J*-*V* characteristics of the best PSC devices based on spiro-OMeTAD as a HTM with carbon and gold counter electrodes.


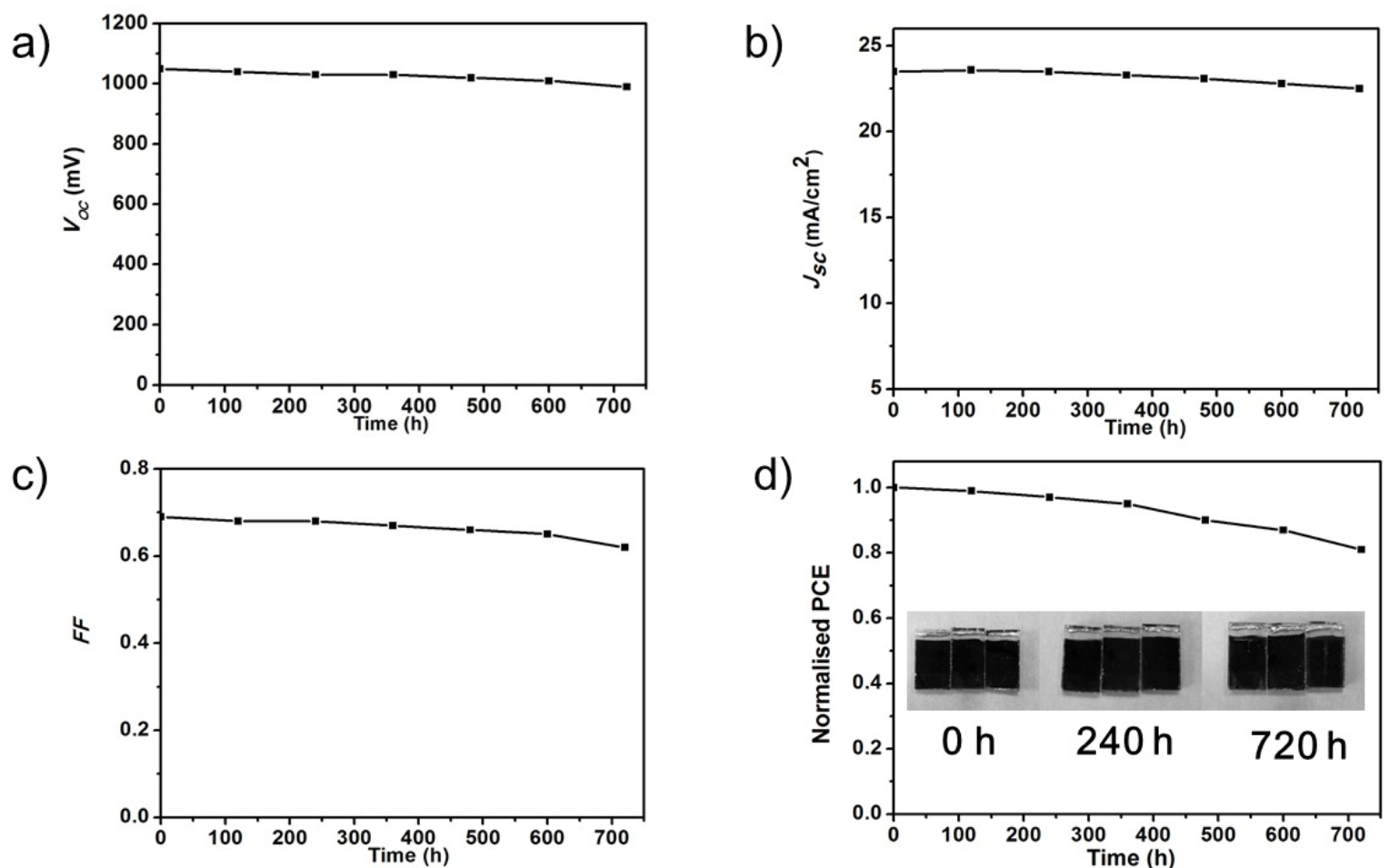


Figure S6. Stability tests for PSC devices based on PEDOT as a HTM. The device without encapsulations were stored at ambient conditions in the dark at room temperatures with a humidity about 30% measured under 100 mW cm^–2^ illumination (AM 1.5G).


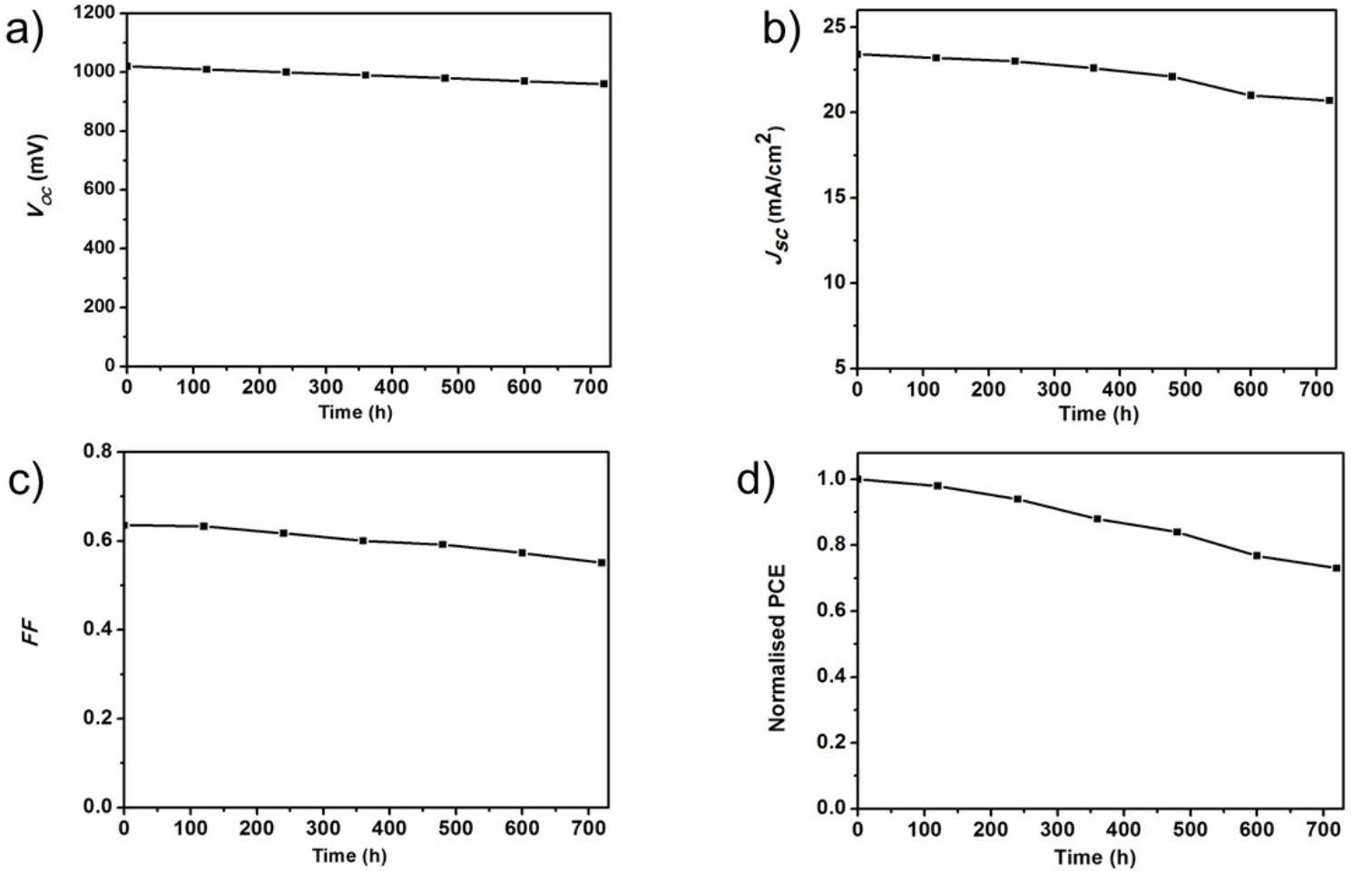


Figure S7. Stability tests for PSC devices based on spiro-OMeTAD as a HTM (containing additives LiTFSI and TBP) with carbon counter electrode. The device without encapsulations were stored at ambient conditions in the dark at room temperatures with a humidity about 30% measured under 100 mW cm^–2^ illumination (AM 1.5G).

**Table S1.** Photovoltaic parameters of PSCs based on spiro-OMeTAD as a HTM (containing additives LiTFSI and TBP) with carbon and gold counter electrodes measured under 100 mW cm^–2^ illumination (AM 1.5G).

| Counter electrode | *V_oc_*  (V) | *J_sc_*  (mA/cm^2^) | *FF* | *PCE*  (%) |
| --- | --- | --- | --- | --- |
| carbon | 1.02 | 23.4 | 0.64 | 15.2 |
| gold | 1.08 | 23.5 | 0.73 | 18.6 |
